# Surgical Outcomes in Children With Primary Congenital Glaucoma: An Eight-Year Experience

**DOI:** 10.7759/cureus.9602

**Published:** 2020-08-07

**Authors:** Asmaa Mohammedsaleh, Lina H Raffa, Nawaf Almarzouki, Rana M Jubran, Ahd Al-Harbi, Alanoud H Alluqmani, Aya Mousa

**Affiliations:** 1 Ophthalmology, King Abdulaziz University Hospital, Jeddah, SAU

**Keywords:** axial length, intraocular pressure, retrospective studies, saudi arabia

## Abstract

Background

Primary congenital glaucoma (PCG) is a congenital anomaly arising from an unusual development of the ﬁltration angle, causing a remarkable rise in intraocular pressure (IOP) that is irrelevant to other ocular or systemic deformities.

Purpose

The aim of the current study was to evaluate the surgical outcome in PCG patients between 2011 and February 2019.

Methods

This was a retrospective study of PCG patients who underwent trabeculotomy, trabeculectomy, deep sclerectomy, Ahmed valve, and/or cyclophotocoagulation (CPC) at a tertiary hospital. The IOP must be equal or less than 21 mmHg with or without medication to be designated a successful surgery.

Results

A total of 80 eyes (41 patients) were included in the study, with a slight male predominance of 65.9%. At presentation, the most reported age group was under 30 days (46.3%). Moreover, deep sclerotomy was the most common procedure, followed by CPC, which were performed in 48 (58.5%) and 18 (21.9%) eyes, respectively. In the overall group, the mean initial IOP was 23.65±8.52 mmHg, while the mean IOP was 16.73±8.56 mmHg at final follow-ups (p < 0.001), with a 46% reduction. The mean axial length showed a slight progression from 21.11±2.58 at the initial visit to 22.92±3.57 mm at the last follow-up (p<0.001). However, the mean horizontal diameter increased from 12.63±1.83 mm at the initial visit to 13.31±1.13 mm at the final visit (p=0.004).

Conclusion

An excellent IOP reduction could be accomplished in the majority of eyes. Deep sclerectomy can effectively reduce IOP in PCG without the occurrences of serious complications that are commonly seen after other procedures like trabeculectomy or trabeculotomy.

## Introduction

Primary congenital glaucoma (PCG) is a congenital anomaly due to autosomal recessive inheritance, manifesting as an unusual development of the ﬁltration angle, causing elevated intraocular pressure (IOP) independently of other ocular or systemic disorders [1,2]. It is more common in Saudi Arabia due to a higher rate of consanguineous marriages and is responsible for 16% of childhood blindness [2-4].

Delayed diagnosis and treatment of PCG can lead to damage to the optic nerves in children [3]. Moreover, the estimated incidence of congenital glaucoma among ophthalmic patients in Saudi Arabia is 1:2 , whereas it is less common in Western countries, where the incidence is 1:10,000 [4].

Based on several studies, the procedures of choice for treating PCG are trabeculotomy and goniotomy, as higher success rates are achieved by these methods [4,5]. However, the success rates of IOP reduction vary across countries, ranging from 19.4% to 91% [6,7]. In India, Mandal et al. reported that a combined trabeculotomy‒trabeculectomy procedure showed better success rates in 41.1% of PCG patients compared with infantile and juvenile onset glaucoma groups [8]. Reports on comparing different surgical procedures for PCG and their long-term outcomes and efficacy are rare [4,5]. Hence, this study was undertaken to evaluate the outcomes of different glaucoma surgeries in children with PCG. 

## Materials and methods

Study design

This retrospective study included all PCG patients who underwent trabeculotomy, trabeculectomy, deep sclerectomy (DS), Ahmed valve, and/or cyclophotocoagulation (CPC) surgery at King Abdul-Aziz University Hospital (KAUH) Jeddah between 2011 and February 2019. Information on 116 eyes of 58 patients was collected from an institutional database. However, 34 eyes of 17 patients whose IOP results were missing, or who had secondary types of congenital glaucoma (e.g., Sturge‒Weber syndrome), or had no follow-up data were excluded from the study.

Data variables 

The data assembled from the patients’ medical records included the following: sex, age at presentation, laterality of the disease, number and type of surgeries, refraction when feasible, cause of visual impairment, and medical therapy. Initial and final measurements of the following parameters were included: corneal diameter, IOP, central corneal thickness (CCT), axial length (AL), and cup-to-disc ratio (CDR). Changes in these variables were recorded for the overall group (n=80 eyes of 41 patients), and also for subgroups in which the condition was controlled (n=63 eyes) or failed to be controlled (n=17). An average IOP ≤21 mmHg, with or without topical medication, was considered an indication of surgical success.

Diagnostic criteria and surgical intervention 

The diagnosis of PCG was made based on elevated IOP, corneal diameter, and glaucomatous cupping when visualization was feasible. The examination was performed under sedation with chloral hydrate (100 mg/kg for the first 10 kg, and 50 mg per each additional kg) in the operating room for all patients. 

The horizontal corneal diameter was measured using a caliper. The IOP was recorded by using an applanation tonometer (Perkins Tonometer; Clement Clarke International, Ltd., Harlow, UK or Tono-Pen XL Tonometer, Reichert Technologies, Depew, NY). The CDR was evaluated by fundus examination when possible. Additionally, ultrasonography (US-4000, Nidek Co., Ltd., Gamagori, Japan) was performed to measure the AL and CCT. 

Statistical analysis

Data were analyzed using Microsoft Excel and Statistical Package of the Social Sciences Version 21 (IBM Corp, Armonk, NY). Basic descriptive statistics were used to analyze the study variables, including frequency, percentage, and mean and standard deviation. The chi-square test was applied to establish the relationship of IOP control to demographic data and age at surgical intervention. Analysis of variance was used to compare the mean IOP at the final visit and IOP drop between different surgeries. Additionally, an independent t-test was used to evaluate the change in variables from the initial to the final visits between the controlled and failed subgroups. A paired t-test was used to evaluate the differences between continuous variables. Notably, a p value ≤0.05 was considered statistically significant

## Results

Demographic data 

A total of 41 patients (80 eyes) with a slight male predominance (65.9% vs. 34.1%) were studied. The demographic characteristics and data of the PCG cases are shown in Table 1.

**Table 1:** Sociodemographic characteristics and data in PCG PCG, primary congenital glaucoma; IQR, interquartile range.

Characteristics	Category	n	%
Age at presentation	Median 30; IQR (15-165)		
Age group at presentation	<30 days	24	58.5
	30-180 days	8	19.5
	>180 days	9	22
Gender	Male	27	65.9
	Female	14	34.1
Surgery laterality	Bilateral	49	95.1
	Unilateral	2	4.9
Follow-up months	Median 17.16 IQR (3.9-38.21)		

Surgical intervention

The median number of surgeries for the eyes was 2 (IQR=2-3 and 1-3 for the right and left eyes, respectively). The number of eyes of PCG patients who needed a single surgery was 47 (58.7%), while 22 eyes (27.5%) underwent two surgeries, five eyes (6.2%) needed three surgeries, five eyes (6.2%) underwent four surgeries, and only one eye (1.2%) required five surgeries.

The most common surgical intervention used was deep sclerotomy in 74 eyes (60.2%), followed by CPC in 33 eyes (26.8%), Ahmed valve implant in seven eyes (5.7%), trabeculectomy in seven eyes (5.7%), and trabeculotomy in two eyes (1.6%). Postoperative complications included hyphemia in three eyes after trabeculotomy surgery, as well as vitreous hemorrhage after the revision of an Ahmed valve. However, no serious complications were reported. DS was the most common primary procedure with an excellent safety profile.

Intraocular pressure 

**Figure 1:**
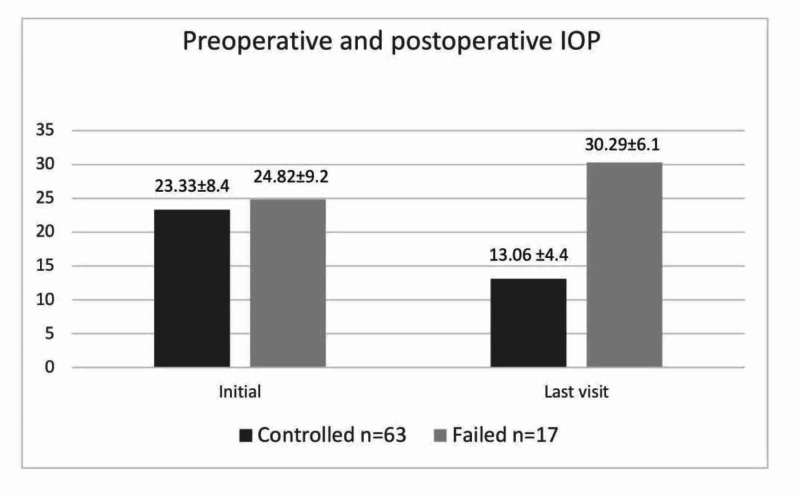
Comparison of variables mean (SD) intraocular pressure changes (mmHg) at initial and final visits between controlled and failed groups IOP, intraocular pressure; SD, standard deviation

In the overall group, the reduction in IOP levels between the initial (23.65±8.52 mmHg) and final visits (16.73±8.56 mmHg) was 46%. The mean initial and final IOP measurements of each subgroup are shown in Figure 1.

In the controlled group, IOP was controlled in 56 of 63 eyes by means of medication, and in seven eyes without medication. Of the overall 80 eyes, 17 were considered to be surgical failures. There was a significant decrease in IOP between the initial and final visits in the controlled group (p<0.001). However, no significant decrease in IOP was found in the failed group (Table 2). The degree of IOP decrease between the controlled and failed subgroups was not statistically significant. Based on the surgical procedures, the difference in the mean IOP drop was greatest in the DS group (-8±10.8), followed by Ahmed valve (-3±9.1), and there was no significant change in the CPC group (0.35±8.5) (p=0.003). The final IOP was lowest in the DS group (15.85±7.3 mmHg), followed by the CPC group (19.28±7.8 mmHg) and Ahmed valve (20±5.1 mmHg) (p=0.129).

**Table 2:** Comparison of variables at initial and final visits between controlled and failed groups AL, axial length; CCT, central corneal thickness; CDR, cup-to-disc ratio; IOP, intraocular pressure

	Controlled (Initial)	Controlled (Final)	P value	Failed (Initial)	Failed (Final)	P value
IOP	23.33±8.38	13.06±4.43	<0.000	24.82±9.16	30.29±6.13	0.058
CDR	0.49±0.41	0.36±0.22	0.057	0.63±0.21	0.76±0.17	0.176
AL	20.80±1.91	22.56±1.47	0.005	23.09±2.76	26.70±2.34	0.009
CCT	710.90±157.59	588.25±93.79	<0.000	790.38±305.62	723.31±284.97	0.027
Corneal diameter	12.40±1.80	13.08±0.95	0.015	13.62±1.65	14.29±1.37	0.082

Cup-to-disc ratio 

**Figure 2:**
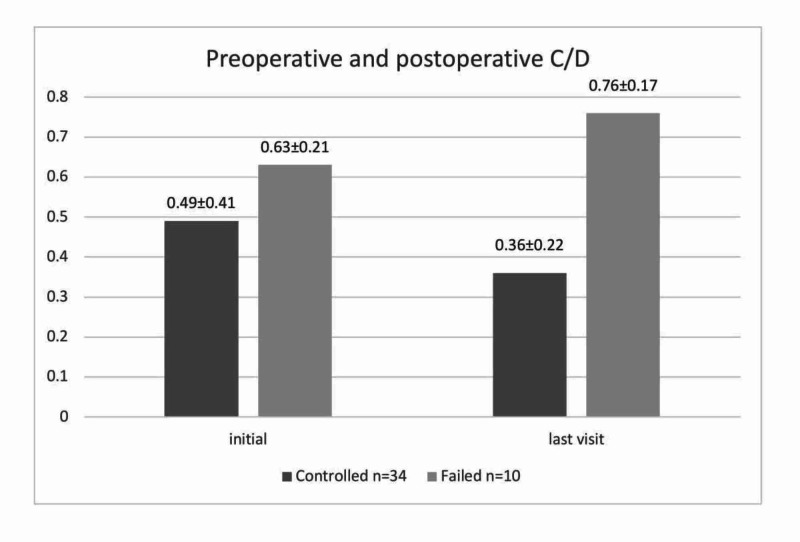
: Comparison of mean (SD) cup-to-disc ratio values at initial and final visits between controlled and failed groups C/D: cup-to-disc ratio; SD, standard deviation

In the overall group, there was no marked change between the initial (0.53±0.37) and final CDR (0.45±0.27) (p>0.05). The mean initial and final CDRs of each subgroup are shown in Figure 2. The changes in CDR were not significant in either subgroup.

Axial length

**Figure 3:**
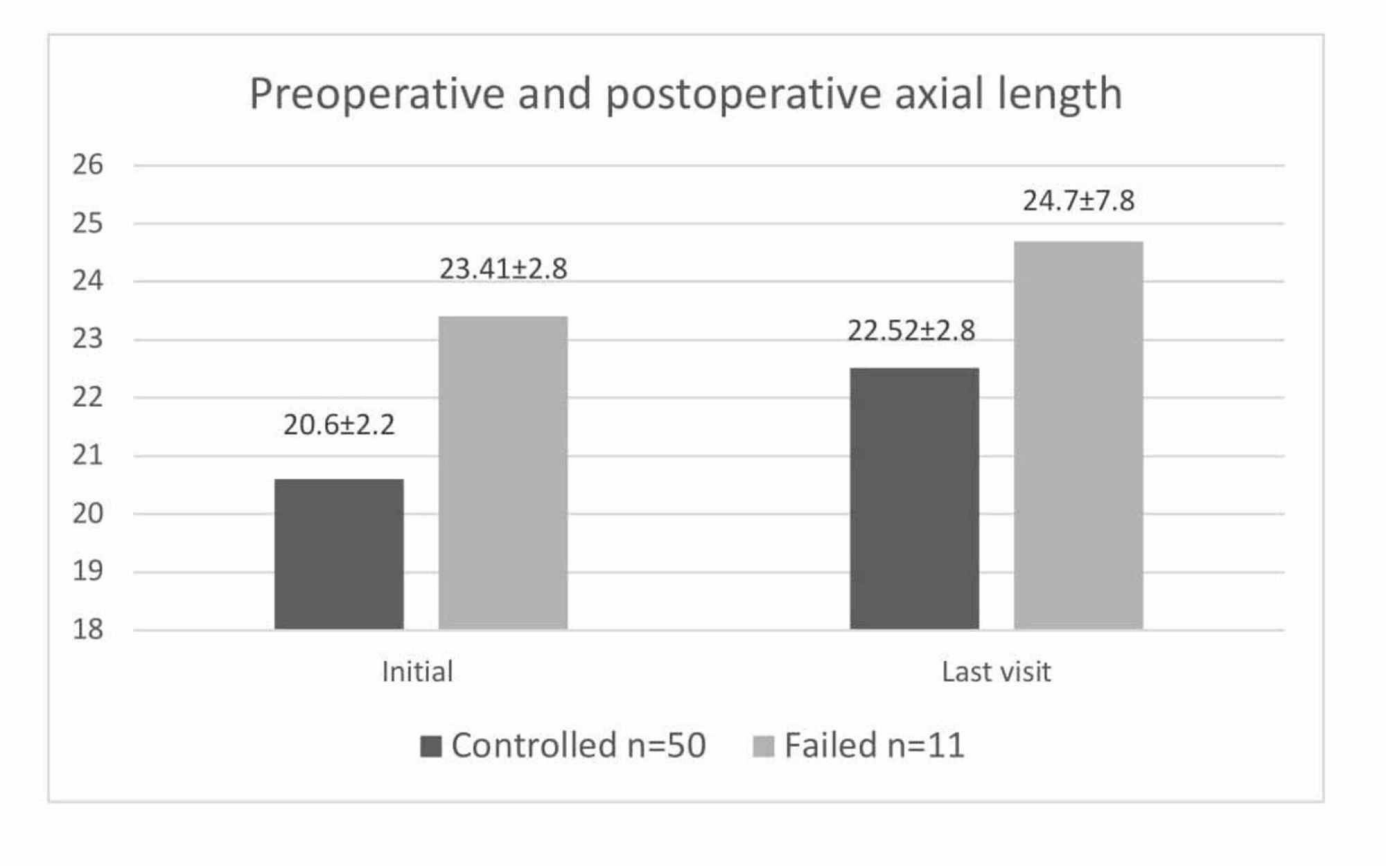
Comparison of mean (SD) axial length changes (mm) at initial and final visits between controlled and failed groups SD, standard deviation

In the overall group, the mean AL showed a slight progression from 21.11±2.58 mm at the initial visit to 22.92±3.57 mm at the final follow-up (p<0.001). The mean AL of each subgroup is shown in Figure 3. Significant changes in AL between the initial and final visits were detected in both the controlled (p=0.005) and failed (p=0.009) subgroups (Table 2). When the degree of axial lengthening was compared, the controlled group showed a smaller increase than the failed group (p=0.002).

Corneal diameter, thickness, and clarity

**Figure 4:**
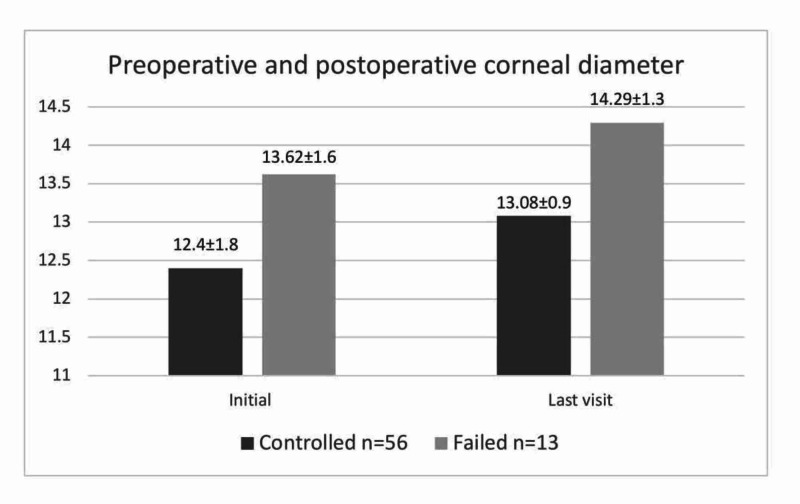
Comparison of mean (SD) corneal diameter changes (mm) at initial and final visits between controlled and failed groups SD, standard deviation

In the overall group, the mean horizontal diameter increased from 12.63±1.83 mm at the initial visit to 13.31±1.13 mm at the final visit (p=0.004). The mean horizontal diameters at the initial and final visit are shown in Figure 4. The increase was significant only in the controlled group (p=0.015; Table 2). However no significant difference was found between the initial and final corneal diameter reading in the failed subgroup. 

**Figure 5:**
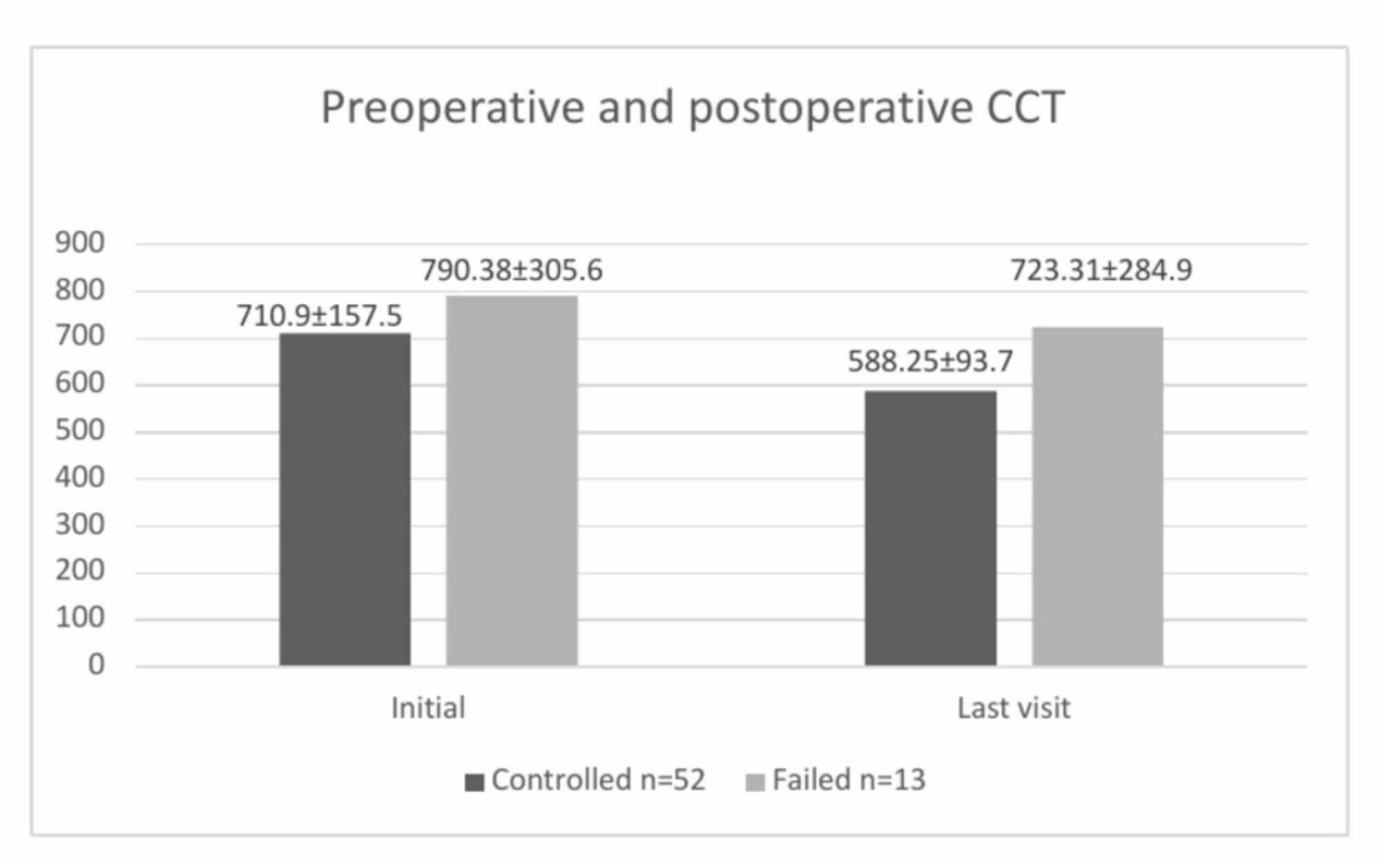
Comparison of mean (SD) central corneal thickness changes (µm) at initial and final visits between controlled and failed groups CCT, central corneal thickness; SD, standard deviation

In the overall group, the mean CCT decreased from 726.8±195.78 μm initially to 615.26±168.75 μm at the final visit (p<0.001). The mean initial and final values of the CCT of each subgroup are shown in Figure 5. The differences between the initial and final visits in both subgroups were found to be significant (Table 2). However, the decreases in CCT were not significantly different in the subgroups. The change in corneal clarity was significant between the initial and final visits (p<0.001) as shown in Table 3. Non-clear cornea included corneas that were hazy, cloudy, or that had haab striae, scaring, and/or edema.

**Table 3:** Corneal clarity at initial and final visits *Total number is less than 80 eyes due to missing documentation

Corneal clarity	Initial visit (n=66)*	Last visit (n=56)*	P value
Clear	19	23	<0.001
Non-clear	47	33	

Success rate

At the final follow-ups, 63 eyes (78.75%) demonstrated surgical success (IOP <21 mmHg), among which the IOP was controlled with and without medication in 56 (88.8%) and 7 eyes (11.1%), respectively. Seventeen eyes (21.2%) were considered surgical failures. IOP control was not significantly correlated with age at presentation, nor was it correlated with sex, or age at surgical intervention. 

## Discussion

In this study with a follow-up period of 6.7 years in 80 eyes, there was a 46% reduction in IOP levels between the initial (23.65±8.52 mmHg) and final visits (16.73±8.56 mmHg). At the final follow-up, 63 eyes (78.75%) demonstrated surgical success (IOP <21 mmHg), while surgical treatment failed in 17 eyes (21.2%). The incidence of PCG was lower in female than in male patients (34.1% vs. 65.9%, respectively). Previous studies on PCG have also revealed a male preponderance with a male to female ratio of 3:1 [6,9-13]. On the other hand, some studies have reported an equivalent distribution between the sexes, while a study from Japan showed a female preponderance (63% females, 37% males) [3,4,14,15].

Most PCG cases presented within the first 30 days of life (46.3%), followed by cases from 30 to 180 days (31.7%), and more than six months of age (22%). Likewise, the age of onset of PCG in the study by Yassin et al. was within the first year after birth with a higher frequency of 87% [5]. In comparison, another study showed a late age of presentation at around eight years, whereas we encountered only three patients who presented after the age of one year [16]. In our study, the frequency of bilateral disease was 95.3%. This was higher than that reported in previous studies (rates of 67.1%, 74%, and 82.6%) [3-5,14,16,17].

In this study, the mean IOP in the controlled group had decreased markedly at the final visit (13.06±4.43 mmHg) as compared to the initial levels (23.33±8.38 mmHg), reflecting an IOP reduction of 46%. This finding is similar to that reported by Yassin et al. and Dubey et al. with IOP reduction of 32.9% and 44%, respectively, after trabeculotomy and combined trabeculotomy‒trabeculectomy procedures [5,18]. In our study, IOP control was achieved in 78.75% of PCG cases with or without medication. More specifically, in 88.88% of cases, IOP was controlled with medication, and in 11.11% it was controlled without medication. A success rate higher than 94%, with and without medication, was reported in the British Infantile and Childhood Glaucoma Eye Study [19]. Yassin et al. reported achieving IOP control in 80.4% of children with PCG, with medication in 64.7% of them and without medication in 35.3% [5].

The levels of initial corneal transparency are categorized into hazy/cloudy, clear, Haab’s striae, and scars [4,5,20]. The most common presentation of corneal transparency at the initial visit in this study was haze, as it was in Yassin et al.’s study [5]. On the other hand, Haab’s striae were most common in Sood et al.’s study [9]. Significant improvement was achieved in corneal clarity postoperatively in our study, similar to results from a study on combined trabeculotomy- trabeculectomy [10].

IOP and AL are essential parameters for deciding on the necessity for repeated surgery, while AL is, in particular, essential for longer-term monitoring [21,22]. The mean AL in the controlled group increased significantly between the initial and final visits; however, a lesser degree of lengthening was detected in the controlled group than in the failed group. Axial lengthening in this study was consistent with findings from previous studies [23]. In many eyes with congenital glaucoma, ALs are longer than expected compared with the same age group; however, ALs eventually reach values similar to those of normal eyes [21,24].

In this study, the CCT in PCG patients was 726.80±195.78 μm at the initial visit and decreased significantly to 615.26±158.75 μm at the final visit. The significant decrease, which was observed even in the failed group, is beneficial although the ideal IOP is not reached post-surgery. Lopes et al. found that the mean CCT at the initial visit was 543.3±66.9 µm in PCG eyes in a study conducted in the United States. In comparison, CCT values were markedly higher preoperatively, possibly depicting a more aggressive pattern in PCG cases from our region [20].

In this study, there was no statistically significant difference in the mean CDR between the initial (0.53±0.37) and final (0.45±0.27) visits (p=0.197). In a study from Germany, an increase in CDR from 0.47±0.25 at the first presentation to 0.73±0.26 at the last follow-up in 18 eyes and a decrease from 0.61±0.24 to 0.39±0.32 in 19 eyes were reported, and the ratio remained unchanged in 24 eyes (0.39±0.32) [23]. According to Zhang et al, the pre- and postoperative mean CDR values in the trabeculotomy group were 0.71±0.24 and 0.67±0.15, respectively, showing no significant difference [5]. Several studies have concluded that the earlier the patient undergoes surgery and achieves successful IOP control, the higher the probability of reversing glaucomatous cupping [7,25].

In the overall group, the mean corneal diameter increased significantly from 12.63±1.83 mm at the initial visit to 13.31±1.13 mm at the final visit. Another study showed no statistical difference in the corneal diameter between the first (13.1±0.9 mm) and last visits (13.4±0.8 mm), and the corneal diameter remained constant after the first year of age [23]. In accordance with our results, a Brazilian study demonstrated a significant increase in the mean corneal diameter from 13.45±1.00 mm preoperatively to 13.98±1.01 mm postoperatively [26].

There were no significant associations between IOP control and sex or age at presentation, or between IOP control and age at surgical intervention in the <six months age group (p=043, p=0.27, respectively). However, in many studies, age >six months was associated with a poor outcome [5, 15]. This is in accordance with a study that reported a 90% chance of IOP control following surgical intervention performed between the age of two months and one year compared with interventions performed in patients of over one year of age [27].

The choice of surgical procedure is dependent on several factors, including the patient’s ocular condition, and the surgeon’s experience and preference. Deep sclerotomy and CPC were used as the first choice of surgeries in our study (58.5% and 21.9%, respectively). Previous studies have shown a remarkable change in IOP before and after DS [28,29]. Gorsler et al. studied the reduction in IOP after CPC in an adult population (-1.55±2.50 mmHg); however, no studies have been conducted in terms of the use of CPC in pediatric PCG patients [30]. In our study, 26 eyes underwent CPC and demonstrated a marked drop in mean IOP (0.35±8.5 mmHg). In our study, less than a third of the patients had marked corneal haze; hence, goniotomy was not our first choice of procedure in contrast to other studies [4]. Moreover, no major complications, such as severe hypotony, choroidal effusion, or endophthalmitis, were documented in this study. In addition, in this cohort, DS had the most significant effect on the final IOP as well as the IOP drop, indicating that it is one of the safest and efficacious options. This is in agreement with the majority of studies on the long-term surgical outcome of PCG [5].

The limitations of this study were insufficient documentation, a lack of patient follow-up, and the lack of assessment of visual acuity in children less than three years of age, as well as the relatively small sample size.

## Conclusions

In this retrospective study, excellent IOP, CDR, and corneal diameter reduction could be achieved in the majority of eyes. DS can effectively reduce IOP in PCG without the occurrences of serious complications that are commonly seen after other procedures like trabeculectomy or trabeculotomy. The use of antiglaucoma medications can lead to more favorable outcomes.
